# Age- and sex- specific all-cause mortality risk greatest in metabolic syndrome combinations with elevated blood pressure from 7 U.S. cohorts

**DOI:** 10.1371/journal.pone.0218307

**Published:** 2019-06-13

**Authors:** Winnie W. Yu, Arshdeep K. Randhawa, Steven N. Blair, Xuemei Sui, Jennifer L. Kuk

**Affiliations:** 1 School of Kinesiology and Health Science, York University, Toronto, Canada; 2 Department of Epidemiology and Biostatistics, Arnold School of Public Health, University of South Carolina, Columbia, SC, United States of America; 3 Department of Exercise Science, Arnold School of Public Health, University of South Carolina, Columbia, SC, United States of America; Ritsumeikan University, JAPAN

## Abstract

**Background:**

The association between metabolic syndrome (MetS) and all-cause mortality is well established but it is unclear if there are differences in mortality risk among the 32 possible MetS combinations. Hence, the purpose of this study is to evaluate the associations between different MetS combinations and its individual components with all-cause mortality, and to examine differences in the association by age and sex.

**Methods:**

A merged sample of 82,717 adults from 7 U.S. cohorts was used.

**Results:**

In our sample, MetS was present in 32% of men, 34% of women, 28% of younger adults (18–65 years) and 62% of older adults (>65 years) with 14,989 deaths over 14.6 ± 7.4 years of follow-up. Risk of all-cause mortality was higher in younger individuals with a greater number of MetS factors present, but in older adults having all 5 MetS factors was the only combination significantly associated with mortality. Regardless of age or sex, elevated blood pressure was the MetS factor most consistently present in MetS combinations that were significantly and most strongly associated with mortality. In fact, elevated blood pressure in the absence of other risk factors was significantly associated with mortality in men (HR, 95% CI = 1.56, 1.33–1.84), women (HR = 1.62, 1.44–1.81) and younger adults (HR = 1.61, 1.45–1.79). Conversely, waist circumference, glucose and triglycerides in isolation were not associated with mortality (p>0.05).

**Conclusion:**

In a large U.S. population, different combinations of MetS components vary substantially in their associations with all-cause mortality. Men, women and younger individuals with MetS combinations including elevated blood pressure had stronger associations with greater mortality risk, with minimal associations between MetS and mortality risk in older adults. Thus, we suggest that future algorithms may wish to consider differential weighting of these common metabolic risk factors, particularly in younger populations.

## Introduction

Metabolic syndrome (MetS) is the clustering of three or more metabolic risk factors as defined by the revised National Cholesterol Education Program (NCEP) criteria [1]. It is well established that individuals with MetS are at an increased risk for CVD [2–4] and all-cause mortality [5,6]. There is heterogeneity in how MetS is defined [7,8] as well as potential sex and age differences in the way metabolic factor combinations are related with mortality risk [9]. To our knowledge, studies that previously identified MetS combinations and their associations with mortality risks had short follow-up time [10] and low incident all-cause mortality associated with each MetS cluster [11]. Thus, few studies have a large enough sample size to categorize participants into each of the 32 MetS combinations and examine the specific mortality risk associated with each combination separately [9]. Hence, the objective of this study is to determine whether different MetS combinations vary in their associations with all-cause mortality and to determine whether there are differences by age and sex.

## Methods

### Study population

Seven U.S. population cohorts were merged to form the study sample including the Aerobics Center Longitudinal Study (ACLS) [12], Atherosclerosis Risk in Communities Study (ARIC) [13], Coronary Artery Risk Development in Young Adults (CARDIA) [14], Cardiovascular Health Study (CHS) [15], Multi-Ethnic Study of Atherosclerosis (MESA) [16], Third National Health and Nutrition Examination Survey (NHANES III) [17,18], and the Continuous NHANES (1999–2008) [19,20]. All study participants provided written informed consent for their respective studies. Complete details of individual study designs, procedures, laboratory measurements clinical examinations and analytic guidelines have been previously reported elsewhere.

Participants were excluded if they had missing information on any of the metabolic risk factor (n = 11,640), age > 90 (n = 10) or had missing mortality follow-up data (n = 246). Participants with missing SBP (n = 207), fasting serum triglycerides >10 mmol/L (n = 104), fasting plasma glucose >30 mmol/L (n = 14) and HDL cholesterol >5 mmol/L (n = 3) were also excluded. The final sample included 82,717 participants.

Limited data access for ARIC, CARDIA, CHS and MESA was obtained through the National Heart, Lung, and Blood Institute (NHLBI) of the National Institutes of Health (NIH). This manuscript was prepared using research materials obtained from the NHLBI Data Repository Information Coordinating Center and does not necessarily reflect the opinions or views of the study survey investigators or the NHLBI. NHANES III and Continuous are available publicly online. Institutional ethics approval to analyze this merged dataset was obtained from York University’s Research Ethics Board (e2013–286 & e2017–364).

### Datasets

**Aerobics Center Longitudinal Study (ACLS)**–these analyses included 37,691 participants over 18 years of age from the original cohort who attended the Cooper Clinic (Dallas, TX) for periodic self- or physician-referred medical examinations between 1987 and 2001. Mortality follow-up to December 31, 2003 was used.

**Atherosclerosis Risk in Communities Study (ARIC)**–these analyses included 14,025 participants aged 44–66 years from the prospective study that began in 1987 that was conducted in four U.S. communities (Washington County, MD; Forsyth County, NC; Jackson, MS; and Minneapolis, MN). Included 14,223 participants aged 44–66 years from the original cohort. Participants were followed semi-annually with mortality follow-up to December 31, 2011 was used.

**Coronary Artery Risk Development in Young Adults (CARDIA)**–these analyses included 4,989 participants aged 18–30 years from the longitudinal study that began in 1985–1986 from Birmingham, AL; Chicago, IL; Minneapolis, MN; and Oakland, CA. Mortality follow-up to December 31, 2011 was used.

**Cardiovascular Health Study (CHS)**–included 4,989 participants aged 65 and older from the longitudinal study that was conducted in 1989–1999 from Sacramento, CA; Hagerstown, MD; Winston-Salem, NC; and Pittsburgh, PA. Annual clinical exams were conducted with semi-annual follow-up to assess health status for incident events and mortality adjudication. Mortality follow-up to December 31, 2011 was used.

**Multi-Ethnic Study of Atherosclerosis (MESA)***–*these analyses included 6,842 ethnically diverse (White, Black, Hispanic and Asian) participants aged 44 to 84 years from the longitudinal study that began in 1999. Participants were followed up every 9–12 months to ascertain medical events. Mortality status was followed through to exam 5 (August 2015).

**National Health and Nutrition Examination Survey (NHANES) III and Continuous**–a series of nationally representative cross-sectional surveys collected using a stratified, multistage, probability cluster design. NHANES III was conducted between 1988 and 1994 in 33,994 individuals, ages 2 months and older. NHANES continuous cycles are released biannually 1999–2000 (n = 9,965), 2001–2 (n = 11,039), 2003–4 (n = 10,122), 2005–6 (n = 9,950), 2007–8 (n = 9,762). Of these, 6,803 participants aged 20–90 years from NHANES III and 6,748 participants aged 18–85 years from NHANES continuous are included in these analyses. Public access Mortality Linkage data file with follow-up through December 31, 2011 was used.

### Survey methods

Age, sex, ethnicity (white), self-reported medical history and medications were assessed by questionnaire. Waist circumference was assessed by trained technicians. Mortality status was established from associated Death Certificate Forms (DTH version), National Death Indexes (NDI) or through follow-up exams (AFU) depending on the individual study.

The 2005 revised National Cholesterol Education Program (NCEP) metabolic syndrome criteria was used to evaluate the association between all 32 metabolic syndrome combinations with all-cause mortality. Metabolic syndrome was defined as having three or more of the following metabolic risk factors: 1) waist circumference ≥ 102cm in men or ≥ 88cm in women, 2) fasting serum triglyceride ≥ 1.7mmol/L or dyslipidemia medication, 3) HDL cholesterol < 1.03mmol/L in men or < 1.3mmol/L in women or dyslipidemia medication 4) systolic blood pressure (BP) ≥ 130mmHg or diastolic BP ≥ 85mmHg or on antihypertensive medication, 5) fasting plasma glucose ≥ 5.6mmol/L or diabetes medication [1].

### Statistical analysis

Hazards ratio (HR) with 95% confidence intervals for all-cause mortality was determined using Cox proportional hazards regression models. Significant interaction of age and sex on mortality was accounted for by stratifying for age (18–65 years and >65 years) and sex. Models were adjusted for age and/or sex, white ethnicity and study cohorts. All analyses allowed random intercepts to account for variation between study samples. Statistical analyses were performed using the SAS statistical software (version 9.4; SAS Institute, Cary, NC) with a Bonferroni adjusted significance defined at p<0.001.

## Results

The proportion of participants with MetS was 32.3% in men and 34.0% in women. In younger adults, 27.8% had MetS whereas 62.1% of older adults had MetS. **Table 1** shows the characteristics of participants stratified by sex, age and MetS status. MetS combinations are categorized by the number of MetS risk factors present. Participant characteristics stratified by each of the 32 MetS combinations is shown in Appendix 1. Clustering of all five risk factors was the most prevalent MetS combination present in 13.2% of men and 17.2% of women with MetS. In men, elevated BP was the most common MetS component (49.4%) whereas elevated waist circumference (45.6%) was the most prevalent in women. The least prevalent component was elevated waist circumference (26.2%) in men and elevated triglycerides (26.9%) in women. In both younger and older adults with MetS, displaying all five metabolic factors was the most prevalent MetS combination (13.7%, 17.8%, respectively). Elevated BP remained the most prevalent MetS component in both younger (41.8%) and older adults (78.8%) whereas elevated triglyceride in younger adults (28.6%) and older adults (42.9%) was the least prevalent metabolic components.

**Table 1:** Characteristics of study population categorized by sex or age and MetS status.

	Men		Women		18–65 years (Young)		>65 years (Old)	
N = 82,717	With MetS	Without MetS	With MetS	Without MetS	With MetS	Without MetS	With MetS	Without MetS
*n* (%)	16,141 (32.3)	33,815 (67.7)	11,137 (34.0)	21,624 (66.0)	19,511 (27.8)	50,702 (72.2)	7,767 (62.1)	4,737 (37.9)
Age (years)	53.9 ± 13.4	44.9 ± 13.9	60.2 ± 13.5	45.0 ± 15.5	49.9 ± 10.0	42.3 ± 12.1	73.1 ± 5.6	73.2 ± 5.9
Men (n, %)	-	-	-	-	12,718 (65.2)	31,217 (61.6)	3,423 (44.1)	2,598 (54.8)
BMI (kg/m^2^)	29.6 ± 4.5	25.5 ± 3.3	30.3 ± 5.9	24.4 ± 4.9	30.6 ± 5.2	25.1 ± 4.0	28.3 ± 4.5	24.9 ± 3.8
White ethnicity (n, %)	13,412 (83.1)	27,691 (81.9)	6,825 (61.3)	15,508 (71.7)	14,708 (75.4)	39,876 (78.7)	5,529 (71.2)	3,323 (70.2)
Waist (cm)	104.2 ± 11.4	90.8 ± 9.7	100.9 ± 14.0	80.4 ± 13.8	104.1 ± 12.7	86.5 ± 12.6	99.8 ± 12.0	89.8 ± 11.5
SBP (mmHg)	132 ± 17	120 ± 15	136 ± 22	115 ± 17	130 ± 18	117 ± 15	142 ± 20	132 ± 21
DBP (mmHg)*	82 ± 12	77 ± 10	73 ± 12	71 ± 10	83 ± 11	75 ± 10	72 ± 12	71 ± 12
Triglycerides (mmol/L)	2.3 ± 1.2	1.2 ± 0.7	1.9 ± 1.0	1.0 ± 0.5	2.2 ± 1.2	1.1 ± 0.6	1.8 ± 1.0	1.2 ± 0.5
HDL (mmol/L)	0.9 ± 0.3	1.3 ± 0.3	1.1 ± 0.4	1.6 ± 0.4	1.1 ± 0.3	1.4 ± 0.4	0.9 ± 0.4	1.3 ± 0.5
Glucose (mmol/L)	6.4 ± 2.0	5.4 ± 0.9	6.4 ± 2.6	5.0 ± 0.8	6.4 ± 2.3	5.2 ± 0.8	6.5 ± 2.3	5.3 ± 1.1
Follow-up Time (years)	13.0 ± 6.9	14.7 ± 7.2	13.9 ± 7.0	15.8 ± 7.8	14.4 ± 7.1	15.6 ± 7.5	11.0 ± 6.0	10.5 ± 5.8
Medication Use (n, %)^†^	2,652 (16.5)	1,095 (3.2)	3,398 (30.7)	848 (3.9)	1,989 (10.3)	1,043 (2.1)	4,061 (52.5)	900 (19.1)
All-cause deaths (n, %)	4,442 (27.5)	3,989 (11.8)	4,204 (37.8)	2,354 (10.9)	3,812 (19.5)	4,047 (8.0)	4,834 (62.2)	2,296 (48.5)

Data are mean ± SD or n, percent (%). MetS; metabolic syndrome, BMI; body mass index, SBP; systolic blood pressure, DBP; diastolic blood pressure, HDL; high-density lipoprotein

*Sample size for DBP: Men: n = 43,499; Women: n = 25,108; Young: n = 56,176; Old: n = 12,431

^†^Sample size for medication use: Men: n = 49,820; Women n = 32,637; Young n = 69,992; Old n = 12,465

**Fig 1:**
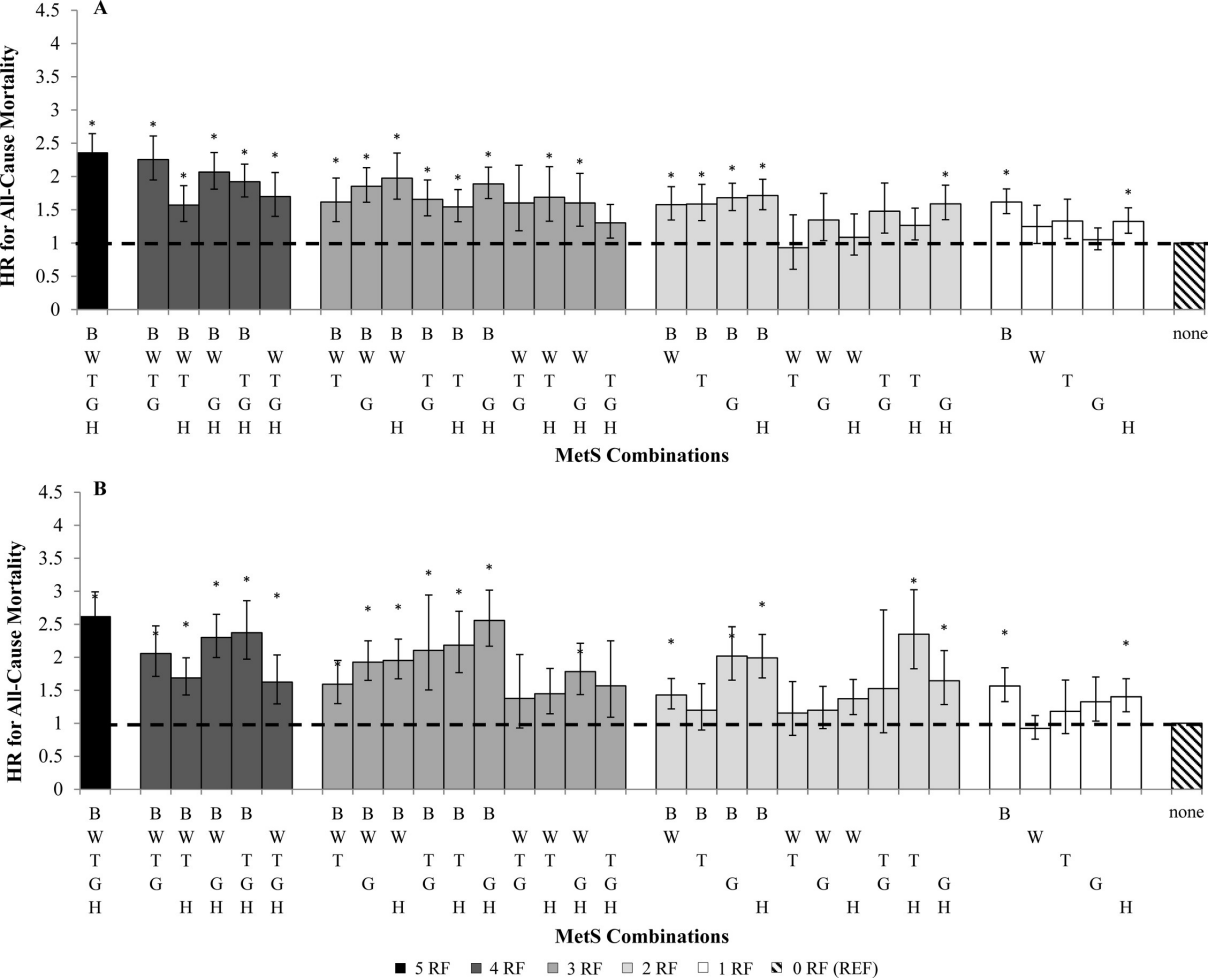
Sex- specific hazards ratio (HR) for all-cause mortality categorized by number of MetS components. (A) men, (B) women. HR analyses are adjusted for age, study, and ethnicity (white). *p< 0.001 of HR compared with zero MetS components (referent). B, blood pressure; W, waist circumference; T, triglyceride; G, glucose; H, HDL; RF, risk factor.

**Fig 2:**
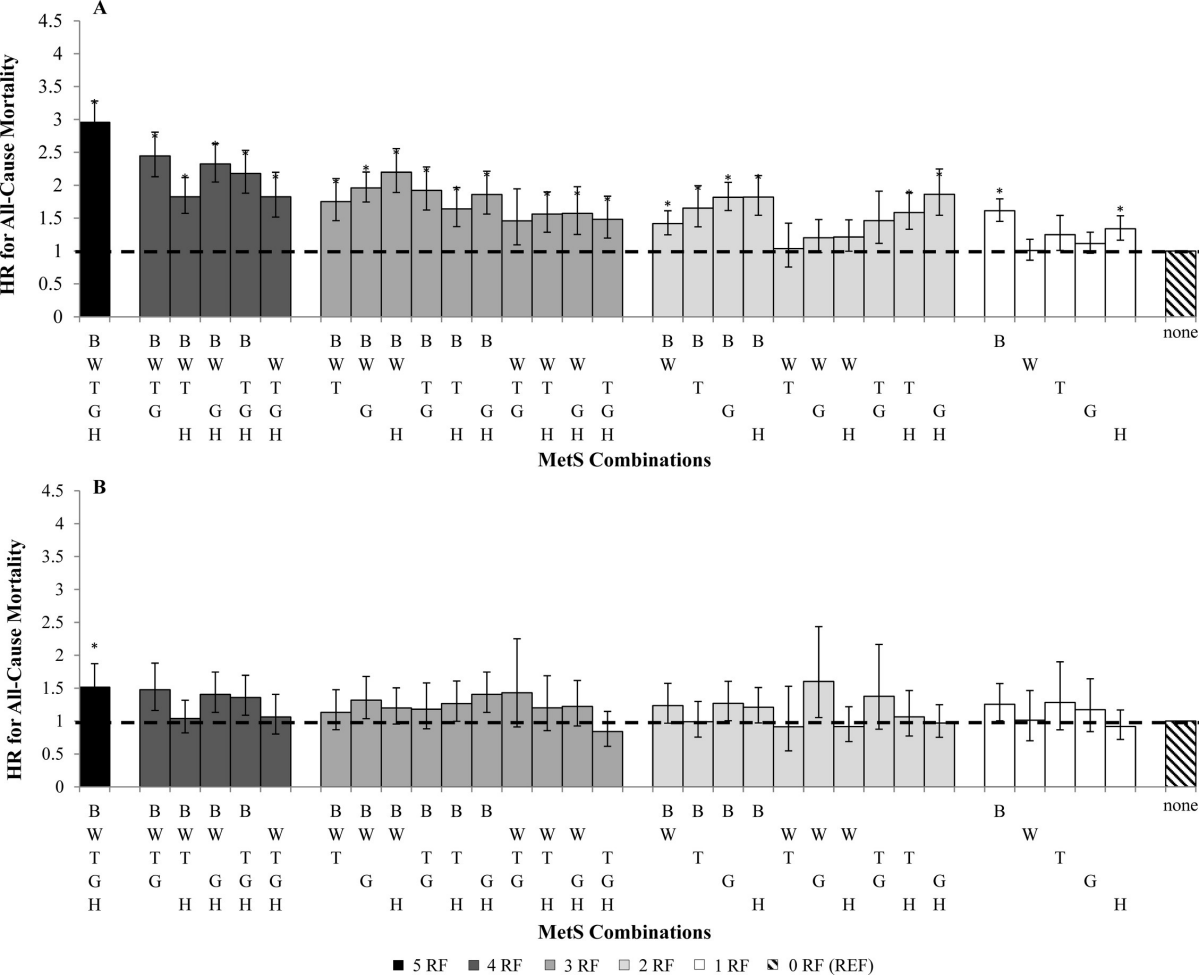
Age- specific hazards ratio (HR) for all-cause mortality categorized by number of MetS components. (A) age 18–65, (B) age >65. HR analyses are adjusted for age, sex, study, and ethnicity (white). *p< 0.001 of HR compared with zero MetS components (referent). B, blood pressure; W, waist circumference; T, triglyceride; G, glucose; H, HDL; RF, risk factor.

There were 14,989 (18.1%) recorded deaths in which 8,646 had MetS during the 14.6 ± 7.4 years mean follow-up period. When adjusted for age, white ethnicity, and study cohorts, women (HR, 95% CI = 1.45, 1.38–1.54, p< 0.0001) and men (HR = 1.32, 1.26–1.38, p< 0.0001) with MetS had a higher risk for all-cause mortality when compared to individuals without MetS. In the adjusted model, younger adults were associated with higher all-cause mortality risk (HR = 1.55, 1.48–1.62, p< 0.0001) than older adults (HR = 1.19, 1.13–1.25, p< 0.0001). Sex- and age- specific hazards ratios of each MetS combination on all-cause mortality are shown in **Fig 1** and **Fig 2**, respectively.

Different combinations of MetS components varied substantially in their associations with mortality. Significant HRs for all-cause mortality were seen in 21 of the 32 MetS combinations in men (HR = 1.54 to 2.36, p< 0.0001) and 20 of 32 MetS combinations in women (HR = 1.43 to 2.62, p< 0.0001). Twenty-three MetS combinations in younger adults were significantly associated with mortality (HR = 1.42 to 2.96, p< 0.001) whereas only having all 5 MetS factors was the only combination associated with mortality in older adults (HR = 1.51, 1.23–1.87, p< 0.0001). Among the MetS combinations with significant associations, having more MetS factors generally was associated with greater HRs for all-cause mortality in men (HR = 1.15, 1.13–1.17, p<0.0001), women (HR = 1.17, 1.15–1.20, p<0.0001), and younger adults (HR = 1.21, 1.19–1.23, p<0.0001). However, a greater number of MetS factors was less strongly related with mortality risk in older adults (HR = 1.08, 1.06–1.11, p<0.0001).

With respects to MetS combinations, the presence of all 5 MetS risk factors in men and women had the strongest association with mortality (Men: HR = 2.46, 2.18–2.78; Women: HR = 2.62, 2.29–2.99, p< 0.0001). Similarly, both young and older adults with all 5 MetS factors had the strongest association with mortality (Young: HR = 2.96, 2.67–3.28, p< 0.0001; Old: HR = 1.51, 1.23–1.87, p< 0.0001).

Elevated BP in the absence of other risk factors was significantly associated with mortality in men (HR, 95% CI = 1.56, 1.33–1.84), women (HR = 1.62, 1.44–1.81) and younger adults (HR = 1.61, 1.45–1.79). MetS combinations with 3 or more risk factors that included elevated BP were more strongly associated with mortality than MetS combinations without BP in both sex groups and younger adults. Low HDL in the absence of the other risk factors was also significantly associated with mortality in men (HR, 95% CI = 1.56, 1.33–1.84), women (HR = 1.40, 1.17–1.67) and younger adults (HR = 1.32, 1.15–1.53). MetS combinations with HDL tended to be less strongly associated with mortality risk as compared to BP. Waist circumference, glucose or triglycerides in isolation were not associated with all-cause mortality in either sex or age group.

## Discussion

This study demonstrates the heterogeneity of the MetS definition and the variations in MetS combinations in relation to all-cause mortality risk in the U.S. adult population. Results from the analyses provide evidence that the 32 combinations of MetS vary in their associations with mortality by age with only modest differences by sex. In particular, we observe that MetS combinations with elevated BP are more predictive of mortality risk than the other MetS factors in both men and women, and younger populations, with the association between MetS and mortality risk being substantively weaker in older populations.

The association between NCEP-defined MetS and all-cause mortality in the U.S. adult population have been well-established [11,21,22]. However, we demonstrate that risk of mortality associated with having a greater number of metabolic risk factors was greater in younger than older adults. In fact, older individuals showed much more modest differences in mortality risks associated with having zero versus 5 metabolic factors (HR = 1.51 vs 2.96, respectively). In fact, the only MetS combination that was associated with mortality risk was having all 5 MetS factors. Lower predictive value of MetS on mortality risk in older adults may be attributed to physiological decline [23] and greater risk associated with other causes of mortality such as dementia, chronic lower respiratory diseases and pneumonia [24]. On the other hand, MetS diagnostic criteria for BP may have underestimated the mortality risk associated with having a high BP older adults. It has been observed that BP is more prevalent with age [25]. Age-related thickening and stiffness of arteries may have also incorrectly contribute to the higher prevalence of BP seen in this age group [26,27]. Thus, age-specific diagnostic guideline especially for BP may be needed to improve the predictive ability of BP in older populations.

Currently, MetS guidelines have sex-specific cut-offs for denoting elevated WC and HDL but not glucose, BP or triglycerides. It has been previously reported that MetS is more strongly associated with elevated mortality risk in women than men [28–30]. However, previous studies did not examine sex differences in how each of the 32 MetS factor combinations relate with mortality. The present large sample size allows us to extend previous observations and surprisingly, although there were clear sex differences in the prevalence of certain MetS factor combinations, there were only subtle sex differences in how the MetS combinations were associated with mortality risk, with women having a subtly higher risk of mortality with MetS overall. Thus, although we and others [9] observed sex differences in how MetS as a whole relates with mortality, there are no specific MetS combinations that are responsible for this increased mortality risk in women.

Findings also show that the components of MetS may not contribute equally to elevated risk of all-cause mortality. Unlike clinically-defined diabetes, triglycerides, and hypertension [31], elevated levels of WC, glucose and triglycerides in isolation failed to predict mortality risk. That a single pre-clinically elevated risk factor in isolation is not necessarily associated with mortality risk is in line with the concept of the metabolic syndrome, wherein a clustering of pre-clinically elevated risk factors may be necessary for increased risk. Further research is required to determine the optimal weighting of individual risk factors within the MetS definition in a representative population sample.

Previously, it has been observed that MetS combinations with BP as a component have the highest estimated 10-year risk for CVD mortality using the European Society of Cardiology (ESC) risk score [32]. Our results extend these observations and suggest the importance of elevated BP alone or in combination with other MetS factors in predicting mortality regardless of sex. Our observations suggest that BP in isolation is the only metabolic factor associated with all-cause mortality risk and the most prominent factor seen in high risk combinations across our study population. Although reasons behind BP as the strongest risk factor in both men and women are still unclear, it has been suggested that elevated BP and hypertension are directly related to the increase risk of stroke morbidity [33], and CVD deaths [34] which are leading causes of death in the U.S. [35]. Thus, we propose that greater emphasis should be given to the diagnosis and treatment of BP, particularly in younger populations.

There are strengths and limitations of this study that merit discussion. This study was the first to our knowledge to examine mortality risk associated with each of the 32 MetS combinations in both men and women as well as in younger and older adults. Due to our merged sample of several cohorts, there may have been variations in reporting of questionnaire data or assessment of physiological and lifestyle factors in our sample that was not entirely accounted for by our statistical adjustment and this may have influenced our findings. Ethnic variations were not examined as our sample was predominately of white ethnicity. Finally, our study population is not nationally representative sample of the U.S. and thus, do not allow for accurate determination of prevalence rates on metabolic combinations and the populations attributable risk for each of these MetS combinations.

## Conclusions

In a large-scale U.S. sample, the present study provides evidence that MetS is a heterogeneous syndrome wherein the expressions of different metabolic risk factor combinations show substantial variations in its associations with all-cause mortality by age and modestly by sex. Regardless of sex, individuals with elevated BP in isolation or in a combination with other metabolic factors also had greater mortality risk. Thus, we suggest that the metabolic factors within the MetS definition may warrant age- and risk factor-specific weightings to better account for the differences in predictive value of the metabolic risk factors in different age groups.

## Supporting information

S1 Appendix (DOCX)
