# Supplementary material for: Age- and sex- specific all-cause mortality risk greatest in metabolic syndrome combinations with elevated blood pressure from 7 U.S. cohorts
Source: PLoS One. 2019 Jun 13;14(6):e0218307. doi: 10.1371/journal.pone.0218307 (PMC6564014; doi:10.1371/journal.pone.0218307)
Supplement: S1 Appendix — (DOCX) [file pone.0218307.s001.docx]

**Appendix 1. Characteristics of 32 MetS Groups**

|  | 5 Risk Factors | | | 4 Risk Factors | | | | | | | | | | | | | | | 3 Risk Factors | | | | | | | | |
| --- | --- | --- | --- | --- | --- | --- | --- | --- | --- | --- | --- | --- | --- | --- | --- | --- | --- | --- | --- | --- | --- | --- | --- | --- | --- | --- | --- |
|  | **BWTGH** | | | **BWTG** | | | **BWT H** | | | **BW GH** | | | **B TGH** | | | **WTGH** | | | **BWT** | | | **BW G** | | | **BW H** | | |
| *n,*% | 4050 | , | 4.9 | 1792 | , | 2.2 | 1978 | , | 2.4 | 2360 | , | 2.9 | 1887 | , | 2.3 | 1101 | , | 1.3 | 1490 | , | 1.8 | 2450 | , | 3.0 | 1656 | , | 2.0 |
| ACLS (n, %) | 1051 | , | 26.0 | 636 | , | 35.5 | 550 | , | 27.8 | 389 | , | 16.5 | 827 | , | 43.8 | 420 | , | 38.2 | 348 | , | 23.4 | 812 | , | 33.1 | 312 | , | 18.8 |
| ARIC (n, %) | 1083 | , | 26.7 | 345 | , | 19.3 | 368 | , | 18.6 | 726 | , | 30.8 | 293 | , | 15.5 | 245 | , | 22.3 | 216 | , | 14.5 | 982 | , | 40.1 | 395 | , | 23.9 |
| CARDIA (n, %) | 4 | , | 0.1 | 0 | , | 0.0 | 17 | , | 0.9 | 6 | , | 0.3 | 0 | , | 0.0 | 3 | , | 0.3 | 3 | , | 0.2 | 4 | , | 0.2 | 62 | , | 3.7 |
| CHS (n, %) | 835 | , | 20.6 | 1 | , | 0.1 | 169 | , | 8.5 | 882 | , | 37.4 | 453 | , | 24.0 | 104 | , | 9.5 | 0 | , | 0.0 | 6 | , | 0.2 | 392 | , | 23.7 |
| MESA (n, %) | 351 | , | 8.7 | 266 | , | 14.8 | 427 | , | 21.6 | 123 | , | 5.2 | 108 | , | 5.7 | 108 | , | 9.8 | 472 | , | 31.7 | 166 | , | 6.8 | 194 | , | 11.7 |
| NH3 (n, %) | 345 | , | 8.7 | 173 | , | 14.8 | 240 | , | 21.6 | 132 | , | 5.2 | 113 | , | 5.7 | 71 | , | 9.8 | 164 | , | 31.7 | 250 | , | 6.8 | 194 | , | 11.7 |
| NHC (n, %) | 381 | , | 9.4 | 371 | , | 20.7 | 207 | , | 10.5 | 102 | , | 4.3 | 93 | , | 4.9 | 150 | , | 13.6 | 287 | , | 19.3 | 230 | , | 9.4 | 107 | , | 6.5 |
| Age (years) | 59.1 | ( | 12.4) | 58.1 |  | (12.1) | 55.5 |  | (14.2) | 61.4 |  | (12.8) | 58.1 |  | (13.9) | 52.6 |  | (12.7) | 57.3 |  | (13.6) | 56.3 |  | (11.3) | 55.7 |  | (15.9) |
| Men (n, %) | 2131 | , | 52.6 | 1161 | , | 64.8 | 970 | , | 49.0 | 975 | , | 41.3 | 1543 | , | 81.8 | 659 | , | 59.9 | 684 | , | 45.9 | 1368 | , | 55.8 | 572 | , | 34.5 |
| White (n, %) | 3121 | ( | 77.1) | 1229 |  | (68.6) | 1456 |  | (73.6) | 1560 |  | (66.1) | 1587 |  | (84.1) | 871 |  | (79.1) | 1013 |  | (68.0) | 1556 |  | (63.5) | 972 |  | (58.7) |
| BMI (kg/m^2^) | 32.0 | , | 4.8 | 32.0 | , | 4.9) | 31.4 | , | 4.6 | 31.7 | , | 5.3 | 26.1 | , | 2.5 | 31.5 | , | 4.6 | 30.8 | , | 4.6 | 32.0 | , | 5.2 | 31.6 | , | 5.6 |
| Waist (cm) | 109.3 | ( | 11.1) | 109.6 |  | (11.0) | 107.1 |  | (10.3) | 107.5 |  | (11.4) | 92.52 |  | (7.5) | 108.2 |  | (11.0) | 105.5 |  | (10.5) | 108.3 |  | (11.6) | 105.6 |  | (11.4) |
| SBP (mmHg) | 139 | ( | 19) | 139 |  | (18) | 135 |  | (17) | 140 |  | (20) | 137 |  | (18) | 116 |  | (8) | 137 |  | (19) | 138 |  | (19) | 137 |  | (19) |
| DBP (mmHg) * | 79 | ( | 14) | 81 |  | (14) | 80 |  | (12) | 77 |  | (13) | 81 |  | (13) | 73 |  | (9) | 80 |  | (13) | 84 |  | (13) | 79 |  | (13) |
| TRIG (mM) | 2.8 | ( | 1.3) | 2.2 |  | (1.0) | 2.6 |  | (1.2) | 1.3 |  | (0.3) | 2.8 |  | (1.3) | 2.7 |  | (1.2) | 2.0 |  | (0.9) | 1.1 |  | (0.3) | 1.2 |  | (0.3) |
| HDL (mM) | 0.8 | ( | 0.2) | 1.4 |  | (0.3) | 0.9 |  | (0.2) | 0.9 |  | (0.2) | 0.8 |  | (0.2) | 0.9 |  | (0.2) | 1.5 |  | (0.4) | 1.5 |  | (0.3) | 1.0 |  | (0.2) |
| Glucose (mM) | 7.7 | ( | 3.2) | 7.2 |  | (2.7) | 5.1 |  | (0.3) | 6.9 |  | (2.4) | 6.8 |  | (2.4) | 6.7 |  | (2.1) | 5.1 |  | (0.4) | 6.8 |  | (2.4) | 5.1 |  | (0.3) |
| Follow-up (y) | 13.0 | ( | 6.9) | 11.6 |  | (6.5) | 13.5 |  | (6.7) | 14.0 |  | (7.0) | 13.2 |  | (6.9) | 14.1 |  | (6.8) | 11.5 |  | (6.3) | 14.0 |  | (7.4) | 14.6 |  | (7.1) |
| Med Use (n, %) | 1386 | , | 34.4 | 571 | , | 32.2 | 410 | , | 21.0 | 701 | , | 29.9 | 529 | , | 28.1 | 129 | , | 11.7 | 411 | , | 27.9 | 310 | , | 12.8 | 276 | , | 16.7 |
| Deaths (n, %) | 1729 | , | 42.7 | 488 | , | 27.2 | 464 | , | 23.5 | 1201 | , | 50.9 | 708 | , | 37.5 | 234 | , | 21.3 | 260 | , | 17.5 | 727 | , | 29.7 | 596 | , | 36.0 |

Data are mean (SD) or n, %. NH3 = NHANES III; NHC = NHANES Continuous; BMI = body mass index; SBP = systolic blood pressure; DBP = diastolic blood pressure; TRIG = triglyceride; HDL = high-density lipoprotein; Med = Medication.

Total Sample (n = 82,717); * DBP: n=68,607; ^†^ Medication use: n=82,457

**Appendix 1. Characteristics of 32 MetS Groups (continued)**

|  | 3 Risk Factors | | | | | | | | | | | | | | | | | | | | | | | | | | | 2 Risk Factors | | | | | | |
| --- | --- | --- | --- | --- | --- | --- | --- | --- | --- | --- | --- | --- | --- | --- | --- | --- | --- | --- | --- | --- | --- | --- | --- | --- | --- | --- | --- | --- | --- | --- | --- | --- | --- | --- |
|  | **B TG** | | | | **B T H** | | | | **B GH** | | | | **WTG** | | | | **B TG** | | | | **B T H** | | | | **B GH** | | | **BW** | | | **B T** | | | |
| *n,*% | 1305 | , | 1.6 | 1580 | | , | 1.9 | 1893 | | , | 2.3 | 489 | | , | 0.6 | 1346 | | , | 1.6 | 746 | | , | 0.9 | 1155 | | , | 1.4 | 2587 | , | 3.1 | | 1680 | , | 2.0 |
| ACLS (n, %) | 739 | , | 56.6 | 774 | | , | 49.0 | 564 | | , | 29.8 | 211 | | , | 43.2 | 357 | | , | 26.5 | 224 | | , | 30.0 | 740 | | , | 64.1 | 668 | , | 25.8 | | 813 | , | 48.4 |
| ARIC (n, %) | 196 | , | 15.0 | 202 | | , | 12.8 | 253 | | , | 13.4 | 87 | | , | 17.8 | 259 | | , | 19.2 | 230 | | , | 30.8 | 139 | | , | 12.0 | 862 | , | 33.3 | | 127 | , | 7.6 |
| CARDIA (n, %) | 3 | , | 0.2 | 27 | | , | 1.7 | 5 | | , | 0.3 | 1 | | , | 0.2 | 20 | | , | 1.5 | 11 | | , | 1.5 | 5 | | , | 0.4 | 31 | , | 1.2 | | 29 | , | 1.7 |
| CHS (n, %) | 1 | , | 0.1 | 197 | | , | 12.5 | 949 | | , | 50.1 | 0 | | , | 0.0 | 38 | | , | 2.8 | 129 | | , | 17.3 | 81 | | , | 7.0 | 0 | , | 0.0 | | 0 | , | 0.0 |
| MESA (n, %) | 125 | , | 9.6 | 183 | | , | 11.6 | 36 | | , | 1.9 | 67 | | , | 13.7 | 274 | | , | 20.4 | 50 | | , | 6.7 | 61 | | , | 5.3 | 416 | , | 16.1 | | 337 | , | 20.1 |
| NH3 (n, %) | 82 | , | 9.6 | 121 | | , | 11.6 | 62 | | , | 1.9 | 33 | | , | 13.7 | 187 | | , | 20.4 | 41 | | , | 6.7 | 48 | | , | 5.3 | 352 | , | 16.1 | | 156 | , | 20.1 |
| NHC (n, %) | 159 | , | 12.2 | 76 | | , | 4.8 | 24 | | , | 1.3 | 90 | | , | 18.4 | 211 | | , | 15.7 | 61 | | , | 8.2 | 81 | | , | 7.0 | 258 | , | 10.0 | | 218 | , | 13.0 |
| Age (years) | 54.5 |  | (12.5) | 52.7 | |  | (14.4) | 62.9 | |  | (14.6) | 53.4 | |  | (11.6) | 47.3 | |  | (13.4) | 53.0 | |  | (13.8) | 49.0 | |  | (12.2) | 54.9 |  | (13.1) | | 53.1 |  | (14.5) |
| Men (n, %) | 1124 | , | 86.1 | 1245 | | , | 78.8 | 1381 | | , | 73.0 | 334 | | , | 68.3 | 633 | | , | 47.0 | 337 | | , | 45.2 | 1024 | | , | 88.7 | 1126 | , | 43.5 | | 1243 | , | 74.0 |
| White (n, %) | 1006 |  | (77.1) | 1317 | |  | (83.4) | 1628 | |  | (86.0) | 377 | |  | (77.1) | 959 | |  | (71.3) | 585 | |  | (78.4) | 1000 | |  | (86.6) | 1556 |  | (60.2) | | 1249 |  | (74.4) |
| BMI (kg/m^2^) | 25.9 | , | 2.7 | 25.8 | | , | 2.6 | 25.0 | | , | 2.7 | 30.7 | | , | 4.0 | 30.8 | | , | 4.6 | 31.0 | | , | 4.9 | 26.0 | | , | 2.4 | 31.1 | , | 5.1 | | 25.3 | , | 2.8 |
| Waist (cm) | 91.9 |  | (7.4) | 90.9 | |  | (7.9) | 89.2 | |  | (8.6) | 107.0 | |  | (9.5) | 104.7 | |  | (10.0) | 105.0 | |  | (10.2) | 91.9 | |  | (7.4) | 105.0 |  | (11.2) | | 89.1 |  | (8.6) |
| SBP (mmHg) | 136 |  | (17) | 134 | |  | (17) | 140 | |  | (20) | 117 | |  | (8) | 114 | |  | (9) | 115 | |  | (8) | 115 | |  | (8) | 137 |  | (19) | | 134 |  | (16) |
| DBP (mmHg) * | 84 |  | (12) | 83 | |  | (11) | 78 | |  | (13) | 74 | |  | (8) | 71 | |  | (9) | 71 | |  | (9) | 74 | |  | (7) | 82 |  | (12) | | 83 |  | (11) |
| TRIG (mM) | 2.3 |  | (0.9) | 2.6 | |  | (1.2) | 1.2 | |  | (0.3) | 2.2 | |  | (0.8) | 2.5 | |  | (1.0) | 1.3 | |  | (0.3) | 2.7 | |  | (1.2) | 1.1 |  | (0.3) | | 2.1 |  | (0.9) |
| HDL (mM) | 1.3 |  | (0.3) | 0.9 | |  | (0.2) | 0.8 | |  | (0.2) | 1.3 | |  | (0.3) | 0.9 | |  | (0.2) | 0.9 | |  | (0.2) | 0.8 | |  | (0.2) | 1.5 |  | (0.4) | | 1.4 |  | (0.3) |
| Glucose (mM) | 6.6 |  | (2.2) | 5.1 | |  | (0.4) | 6.4 | |  | (1.7) | 6.7 | |  | (2.2) | 5.1 | |  | (0.3) | 6.4 | |  | (1.7) | 6.3 | |  | (1.6) | 5.1 |  | (0.3) | | 5.1 |  | (0.4) |
| Follow-up (y) | 12.4 |  | (6.9) | 13.7 | |  | (7.1) | 13.2 | |  | (7.1) | 12.8 | |  | (6.6) | 14.0 | |  | (6.7) | 15.1 | |  | (7.1) | 14.8 | |  | (6.7) | 14.3 |  | (7.5) | | 11.8 |  | (6.7) |
| Med Use (n, %) | 247 | , | 19.1 | 229 | | , | 14.6 | 566 | | , | 30.0 | 87 | | , | 17.8 | 89 | | , | 6.6 | 31 | | , | 4.2 | 78 | | , | 6.8 | 160 | , | 6.2 | | 270 | , | 16.2 |
| Deaths (n, %) | 251 | , | 19.2 | 364 | | , | 23.0 | 1027 | | , | 54.3 | 73 | | , | 14.9 | 168 | | , | 12.5 | 190 | | , | 25.5 | 166 | | , | 14.4 | 550 | , | 21.3 | | 237 | , | 14.1 |

Data are mean (SD) or n, %. NH3 = NHANES III; NHC = NHANES Continuous; BMI = body mass index; SBP = systolic blood pressure; DBP = diastolic blood pressure; TRIG = triglyceride; HDL = high-density lipoprotein; GLU = glucose; Med = Medication.

Total Sample (n = 82,717); * DBP: n=68,607; ^†^ Medication use: n=82,457

**Appendix 1. Characteristics of 32 MetS Groups (continued)**

|  | 2 Risk Factors | | | | | | | | | | | | | | | | | | | | | | | |
| --- | --- | --- | --- | --- | --- | --- | --- | --- | --- | --- | --- | --- | --- | --- | --- | --- | --- | --- | --- | --- | --- | --- | --- | --- |
|  | **B G** | | | **B H** | | | **WT** | | | **W G** | | | **W H** | | | **TG** | | | **T H** | | | **GH** | | |
| *n,*% | 3714 | , | 4.5 | 2143 | , | 2.6 | 916 | , | 1.1 | 896 | , | 1.1 | 1622 | , | 2.0 | 811 | , | 1.0 | 2001 | , | 2.4 | 1391 | , | 1.7 |
| ACLS (n, %) | 2472 | , | 66.6 | 832 | , | 38.8 | 172 | , | 18.8 | 380 | , | 42.4 | 304 | , | 18.7 | 534 | , | 65.8 | 1135 | , | 56.7 | 845 | , | 60.8 |
| ARIC (n, %) | 718 | , | 19.3 | 266 | , | 12.4 | 111 | , | 12.1 | 334 | , | 37.3 | 384 | , | 23.7 | 74 | , | 9.1 | 202 | , | 10.1 | 187 | , | 13.4 |
| CARDIA (n, %) | 16 | , | 0.4 | 104 | , | 4.9 | 6 | , | 0.7 | 4 | , | 0.5 | 138 | , | 8.5 | 4 | , | 0.5 | 90 | , | 4.5 | 23 | , | 1.7 |
| CHS (n, %) | 15 | , | 0.4 | 695 | , | 32.4 | 0 | , | 0.0 | 0 | , | 0.0 | 120 | , | 7.4 | 0 | , | 0.0 | 59 | , | 3.0 | 200 | , | 14.4 |
| MESA (n, %) | 97 | , | 2.6 | 81 | , | 3.8 | 256 | , | 28.0 | 41 | , | 4.6 | 199 | , | 12.3 | 61 | , | 7.5 | 199 | , | 10.0 | 35 | , | 2.5 |
| NH3 (n, %) | 253 | , | 2.6 | 117 | , | 3.8 | 90 | , | 28.0 | 61 | , | 4.6 | 256 | , | 12.3 | 30 | , | 7.5 | 170 | , | 10.0 | 54 | , | 2.5 |
| NHC (n, %) | 143 | , | 3.9 | 48 | , | 2.2 | 281 | , | 30.7 | 76 | , | 8.5 | 221 | , | 13.6 | 108 | , | 13.3 | 146 | , | 7.3 | 47 | , | 3.4 |
| Age (years) | 51.6 |  | (12.4) | 55.0 |  | (17.6) | 47.3 |  | (15.5) | 50.9 |  | (10.8) | 44.8 |  | (15.4) | 48.8 |  | (11.6) | 44.4 |  | (12.8) | 48.8 |  | (14.9) |
| Men (n, %) | 3083 | , | 83.0 | 1388 | , | 64.8 | 351 | , | 38.3 | 502 | , | 56.0 | 453 | , | 27.9 | 699 | , | 86.2 | 1560 | , | 78.0 | 1059 | , | 76.1 |
| White (n, %) | 3117 |  | (83.9) | 1725 |  | (80.5) | 599 |  | (65.4) | 681 |  | (76.0) | 973 |  | (60.0) | 678 |  | (83.6) | 1665 |  | (83.2) | 1252 |  | (90.0) |
| BMI (kg/m^2^) | 25.1 | , | 2.8 | 24.7 | , | 2.9 | 30.1 | , | 4.4 | 30.3 | , | 4.3 | 30.8 | , | 4.9 | 25.5 | , | 2.6 | 25.4 | , | 2.7 | 25.0 | , | 2.7 |
| Waist (cm) | 88.7 |  | (8.8) | 86.6 |  | (9.2) | 103.7 |  | (9.6) | 104.4 |  | (10.1) | 102.1 |  | (9.8) | 90.5 |  | (8.6) | 89.6 |  | (8.4) | 88.0 |  | (9.1) |
| SBP (mmHg) | 135 |  | (17) | 134 |  | (18) | 113 |  | (9) | 115 |  | (9) | 112 |  | (9) | 115 |  | (9) | 113 |  | (9) | 114 |  | (9) |
| DBP (mmHg) * | 86 |  | (10) | 79 |  | (12) | 68 |  | (11) | 74 |  | (8) | 70 |  | (8) | 74 |  | (8) | 73 |  | (8) | 72 |  | (8) |
| TRIG (mM) | 1.0 |  | (0.3) | 1.1 |  | (0.3) | 2.1 |  | (0.9) | 1.1 |  | (0.3) | 1.1 |  | (0.3) | 2.2 |  | (0.8) | 2.5 |  | (1.0) | 1.1 |  | (0.3) |
| HDL (mM) | 1.5 |  | (0.4) | 0.9 |  | (0.2) | 1.5 |  | (0.3) | 1.4 |  | (0.3) | 1.0 |  | (0.2) | 1.3 |  | (0.3) | 0.9 |  | (0.2) | 0.9 |  | (0.2) |
| Glucose (mM) | 6.3 |  | (1.7) | 5.1 |  | (0.3) | 4.9 |  | (0.4) | 6.2 |  | (1.3) | 5.0 |  | (0.4) | 6.3 |  | (1.8) | 5.1 |  | (0.4) | 6.1 |  | (1.3) |
| Follow-up (y) | 13.5 |  | (7.3) | 14.6 |  | (7.1) | 12.2 |  | (6.1) | 15.7 |  | (7.1) | 16.4 |  | (7.0) | 13.0 |  | (6.8) | 15.2 |  | (7.0) | 15.3 |  | (6.6) |
| Med Use (n, %) | 287 | , | 7.8 | 356 | , | 16.7 | 95 | , | 10.4 | 36 | , | 4.0 | 0 | , | 0.0 | 102 | , | 12.6 | 76 | , | 3.8 | 42 | , | 3.0 |
| Deaths (n, %) | 715 | , | 19.3 | 788 | , | 36.8 | 58 | , | 6.3 | 132 | , | 14.7 | 212 | , | 13.1 | 81 | , | 10.0 | 215 | , | 10.7 | 298 | , | 21.4 |

Data are mean (SD) or n, %. NH3 = NHANES III; NHC = NHANES Continuous; BMI = body mass index; SBP = systolic blood pressure; DBP = diastolic blood pressure; TRIG = triglyceride; HDL = high-density lipoprotein; Med = Medication.

Total Sample (n = 82,717); * DBP: n=68,607; ^†^ Medication use: n=82,457

**Appendix 1. Characteristics of 32 MetS Groups (continued)**

|  | 1 Risk Factor | | | | | | | | | | | | | | | | | | | 0 Risk Factors | | | | |
| --- | --- | --- | --- | --- | --- | --- | --- | --- | --- | --- | --- | --- | --- | --- | --- | --- | --- | --- | --- | --- | --- | --- | --- | --- |
|  | **B** | | | | **W** | | | | **T** | | | | **G** | | | | **H** | | |  | |  | |  |
| *n,*% | 6628 | , | 8.0 | 2510 | | , | 3.0 | 1851 | | , | 2.2 | 4513 | | , | 5.5 | 4885 | | , | 5.9 | 17291 | , | | 20.9 | |
| ACLS (n, %) | 3816 | , | 57.6 | 550 | | , | 21.9 | 868 | | , | 46.9 | 3492 | | , | 77.4 | 2374 | | , | 48.6 | 9482 | , | | 54.8 | |
| ARIC (n, %) | 1055 | , | 15.9 | 805 | | , | 32.1 | 160 | | , | 8.6 | 549 | | , | 12.2 | 470 | | , | 9.6 | 1702 | , | | 9.8 | |
| CARDIA (n, %) | 407 | , | 6.1 | 93 | | , | 3.7 | 87 | | , | 4.7 | 59 | | , | 1.3 | 860 | | , | 17.6 | 2867 | , | | 16.6 | |
| CHS (n, %) | 4 | , | 0.1 | 1 | | , | 0.0 | 0 | | , | 0.0 | 1 | | , | 0.0 | 284 | | , | 5.8 | 3 | , | | 0.0 | |
| MESA (n, %) | 455 | , | 6.9 | 340 | | , | 13.6 | 361 | | , | 19.5 | 62 | | , | 1.4 | 160 | | , | 3.3 | 731 | , | | 4.2 | |
| NH3 (n, %) | 537 | , | 6.9 | 359 | | , | 13.6 | 149 | | , | 19.5 | 165 | | , | 1.4 | 424 | | , | 3.3 | 1374 | , | | 4.2 | |
| NHC (n, %) | 354 | , | 5.3 | 362 | | , | 14.4 | 226 | | , | 12.2 | 185 | | , | 4.1 | 313 | | , | 6.4 | 1132 | , | | 6.6 | |
| Age (years) | 48.2 |  | (14.4) | 46.6 | |  | (13.3) | 46.2 | |  | (13.5) | 45.3 | |  | (11.1) | 39.2 | |  | (14.6) | 39.2 |  | | (12.7) | |
| Men (n, %) | 4695 | , | 70.8 | 807 | | , | 32.2 | 1252 | | , | 67.6 | 3451 | | , | 76.5 | 2503 | | , | 51.2 | 9643 | , | | 55.8 | |
| White (n, %) | 5108 |  | (77.1) | 1641 | |  | (65.4) | 1433 | |  | (77.4) | 4111 | |  | (91.1) | 3850 | |  | (78.8) | 13561 |  | | (78.4) | |
| BMI (kg/m^2^) | 24.4 | , | 3.0 | 29.7 | | , | 4.6 | 24.6 | | , | 2.8 | 24.4 | | , | 2.7 | 23.9 | | , | 3.1 | 23.3 | , | | 2.9 | |
| Waist (cm) | 85.2 |  | (9.8) | 101.1 | |  | (9.7) | 86.3 | |  | (9.4) | 85.4 | |  | (9.9) | 81.8 | |  | (10.4) | 79.8 |  | | (10.3) | |
| SBP (mmHg) | 132 |  | (16) | 113 | |  | (9) | 112 | |  | (9) | 113 | |  | (9) | 110 | |  | (9) | 110 |  | | (9) | |
| DBP (mmHg) * | 84 |  | (10) | 71 | |  | (9) | 72 | |  | (8) | 74 | |  | (7) | 70 | |  | (8) | 71 |  | | (8) | |
| TRIG (mM) | 1.0 |  | (0.3) | 1.0 | |  | (0.3) | 2.0 | |  | (0.8) | 1.0 | |  | (0.3) | 1.0 | |  | (0.3) | 0.9 |  | | (0.3) | |
| HDL (mM) | 1.5 |  | (0.4) | 1.5 | |  | (0.4) | 1.4 | |  | (0.3) | 1.5 | |  | (0.3) | 1.0 | |  | (0.2) | 1.5 |  | | (0.4) | |
| Glucose (mM) | 5.1 |  | (0.4) | 5.0 | |  | (0.4) | 5.0 | |  | (0.4) | 6.0 | |  | (1.0) | 4.9 | |  | (0.4) | 4.9 |  | | (0.4) | |
| Follow-up (y) | 13.9 |  | (7.7) | 15.8 | |  | (7.2) | 13.2 | |  | (7.1) | 14.5 | |  | (7.1) | 17.4 | |  | (7.1) | 16.2 |  | | (7.8) | |
| Med Use (n, %) | 168 | , | 2.6 | 0 | | , | 0.0 | 156 | | , | 8.4 | 195 | | , | 4.3 | 0 | | , | 0.0 | 0 | , | | 0.0 | |
| Deaths (n, %) | 988 | , | 14.9 | 240 | | , | 9.6 | 131 | | , | 7.08 | 311 | | , | 6.9 | 498 | | , | 10.2 | 889 | , | | 5.1 | |

Data are mean (SD) or n, %. NH3 = NHANES III; NHC = NHANES Continuous; BMI = body mass index; SBP = systolic blood pressure; DBP = diastolic blood pressure; TRIG = triglyceride; HDL = high-density lipoprotein; Med = Medication.

Total Sample (n = 82,717); * DBP: n=68,607; ^†^ Medication use: n=82,457.
